# Severe Hemorrhage after Tooth Extraction Treated by Transfusion

**Published:** 1883-06

**Authors:** 


### SEVERE HEMORRHAGE AFTER TOOTH EXTRACTION TREATED BY TRANSFUSION.

The *Revue Odontologique* for April contains an interesting account of a case of almost fatal hemorrhage after tooth-extraction. The patient, a young soldier of twenty-two, with a marked history of hereditary and collateral hemorrhagic diathesis, was admitted to the Hotel Dieu, and had some molar roots removed without telling the house surgeon any facts as to his history, and the operation which was performed was followed by a profuse hemorrhage of a dark color, without clots. Next morning plugging with lint and perchloride of iron was tried without permanent effect. On the third day actual cautery was tried at the bottom of the socket, followed by plugging with compressed sponge, the jaws being fixed by a bandage, and ergotine subcutaneously injected. On the fourth and fifth there was no hemorrhage; injections continued. Next day, the sixth, the bandages &c. were removed, owing to sloughing and suppuration of the gums, and from the raw surfaces profuse bleeding recurred, and no local measures were effective to arrest it. On the eleventh day the patient was moribund, and it was decided to try transfusion of blood. After plugging the socket again, 100 grammes of blood were transfused into the cephalic vein, with immediate relief to the patient. In three hours the trouble began again and continued until next morning, when, after a second transfusion, the patient began to revive, although an access of syncope nearly proved fatal during the operation. However, the hemorrhage was stopped, and in six weeks the patient was discharged cured.

